# Migraine prevalence in patients with unruptured intracranial aneurysms: A case–control study

**DOI:** 10.1002/brb3.662

**Published:** 2017-03-30

**Authors:** Elbert H. Witvoet, Nadine Pelzer, Gisela M. Terwindt, Gabriël J.E. Rinkel, Monique H.M. Vlak, Ale Algra, Marieke J.H. Wermer

**Affiliations:** ^1^ Department of NeurologyLeiden University Medical CenterLeidenThe Netherlands; ^2^ Department of Neurology and NeurosurgeryBrain Center Rudolf MagnusUniversity Medical Center UtrechtUtrechtThe Netherlands; ^3^ Department of NeurologyHaaglanden Medical CenterThe HagueThe Netherlands; ^4^ Department of Clinical EpidemiologyLeiden University Medical CenterLeidenThe Netherlands

**Keywords:** intracranial aneurysms, migraine, stroke, subarachnoid hemorrhage

## Abstract

**Objectives:**

Migraine is a suggested risk factor for aneurysmal subarachnoid hemorrhage (aSAH). An increased risk of aSAH in migraineurs may be explained by an increased prevalence of unruptured intracranial aneurysms (UIA). We performed a case–control study to compare lifetime migraine prevalence in patients with UIA, patients with a history of transient ischemic attact (TIA) or ischemic stroke and controls without a history of aSAH, TIA or ischemic stroke.

**Materials and Methods:**

Patients with UIA were recruited from two university hospitals. Data on patients with TIA/stroke were retrieved from a previous study. Partners of patients with UIA or TIA/stroke were included as controls. Migraine history was assessed via a telephone interview based on the International Classification of Headache Disorders, second edition criteria. We calculated odds ratios (OR) for migraine with univariable and multivariable logistic regression analyses, adjusted for age, sex, hypertension and smoking.

**Results:**

We included 172 patients with UIA, 221 patients with TIA or stroke, and 164 controls. In UIA patients, migraine prevalence was 24.4% compared with 14.6% in controls (UIA vs. controls; OR 1.9; 95% confidence interval [CI] 1.1–3.5) and 22.2% in TIA/stroke patients (UIA vs. TIA/stroke; OR 1.1; 95% CI 0.7–1.8). After adjustments, the OR for migraine in UIA patients versus controls were 1.7 (95% CI 1.0–3.1) and 0.9 (95% CI 0.5–1.0) versus TIA/stroke. Results were comparable for migraine with and without aura.

**Conclusions:**

Migraine prevalence is possibly increased in patients with UIA compared with controls and comparable with the prevalence in patients with TIA or stroke. Further studies are needed to confirm our findings and to investigate the underlying pathophysiology.

## 1 Introduction

Migraine, especially migraine with aura, is an established risk factor for ischemic stroke (Etminan, Takkouche, Isorna, & Samii, 2005; Kurth, 2010; Schurks et al., 2009). A recent meta‐analysis of four cohorts and four case–control studies including 1,600 patients with hemorrhagic stroke, revealed a statistically significant increased risk for hemorrhagic stroke in patients with migraine (Sacco, Ornello, Ripa, Pistoia, & Carolei, 2014). Although seven of these eight studies included patients with aneurysmal subarachnoid hemorrhage (aSAH), a subanalysis for hemorrhagic stroke subtype was not possible because of the unavailability of separate data on aSAH and intracerebral hemorrhage. One case–control study with only aSAH patients found no difference in self‐reported migraine prevalence (Carter, Anderson, Jamrozik, Hankey, & Anderson, 2005). The large prospective of Women's Health Study, however, suggested an increased risk of aSAH for women with migraine after long‐term follow‐up although this was not statistically significant (Kurth, Kase, Schurks, Tzourio, & Buring, 2010). Another large prospective population‐based cohort study from Taiwan found that migraine with and without aura were linked to an increased risk of hemorrhagic stroke (Kuo et al., 2013). Among the total number of hemorrhagic stroke patients in this study, the proportion of aSAH was higher in migraine patients compared with controls, indicating an increased risk for aSAH in patients with migraine.

A higher aSAH risk in migraineurs might be explained by a higher prevalence of aneurysms, a higher risk of rupture or both. Possibly, vascular mechanisms involved in the pathophysiology of migraine or associated vascular risk factors such as smoking or hypertension may affect vessel wall integrity (Juvela, Poussa, & Porras, 2001). It is already known that migraine, especially migraine without aura, is associated with the occurrence of cervical artery dissections (Metso et al., 2012). In this study, we investigated the prevalence of migraine with or without aura in patients with unruptured intracranial aneurysms (UIA) compared with patients with TIA/Stroke and with controls.

## 2 Materials and Methods

### 2.1 Patients and controls

Patients with UIA were recruited from a prospectively collected database between 2009 and 2013 at Leiden University Medical Center (LUMC), and from a cohort of participants of a prospective study on risk factors for UIA at University Medical Center Utrecht collected between 2006 and 2009 (Vlak, Rinkel, Greebe, & Algra, 2013). Patients older than 18 years of age were included who had one or more unruptured intracranial aneurysm confirmed by CT/MR‐angiography or conventional angiography. For all UIA patients we recorded the reason for cerebral imaging leading to the detection of the UIA. Patients who were diagnosed with an UIA after cerebral imaging because of headache were excluded, to prevent overestimation of migraine prevalence by selection bias. Patients with a history of aSAH or treatment for their UIA were also excluded, as headache complaints resulting from the aSAH or from treatment may be confused with migraine complaints, and cognitive impairments resulting from the aSAH may interfere with performing a reliable interview.

Patients with TIA/stroke were included to compare patients with an UIA with a group that is known to have a higher prevalence of migraine. TIA was diagnosed by a stroke neurologist on clinical grounds. Patients with a history of TIA or stroke were interviewed in a previous study, in which the questionnaire used in our study was validated. This validation study used exactly the same approach for diagnosing migraine as adapted in patients with UIA and controls in our study. The validation cohort was a random sample of 300 of the 492 patients who were admitted with a TIA or stroke between January 2011 and April 2012 in our hospital. All participants of the validation study completed the migraine screener during their hospital stay. Of these 300 participants, 221 could be contacted by telephone for an interview to verify the migraine diagnosis (van der Willik, Pelzer, Algra, Terwindt, & Wermer, 2017).

Partners of patients with UIA or a history of TIA/stroke were asked to participate as controls. All controls with a history of TIA, ischemic stroke, aSAH or UIA were excluded. The Medical Ethics Committee reviewed the research protocol and granted a waiver for the project.

### 2.2 Migraine interview

All eligible patients with UIA received an invitation letter announcing an interview by telephone. Patients who did not want to be contacted had the possibility to return a statement that they did not want to participate. The interviewer was a medical student (EHW), who was trained to perform a migraine interview by a migraine research physician (NP) from the LUMC. Migraine history was first assessed with the short Migraine Screener for Stroke (MISS) questionnaire (van der Willik et al., 2017). The MISS contains five questions about the self‐diagnosis of migraine, the diagnosis of migraine by a physician, the frequency of attacks, as well as associated symptoms such as vomiting, photo‐ and phonophobia, and concomitant visual symptoms. The MISS has a high negative predictive value (0.99), and a reasonable positive predictive value (0.71). A more detailed interview was conducted when results of the MISS questionnaire were suggestive of migraine (a positive answer to at least one of the questions). In this interview, the migraine diagnosis was verified according to the criteria of the International Classification of Headache Disorders, second edition (ICHD‐2) by the International Headache Society which are comparable with the recent updated ICHD‐III criteria (Headache Classification Committee of the International Headache Society, 2013). All interviews in patients and controls were discussed with a migraine research physician (NP) to confirm the diagnosis. In case of ambiguity a headache specialist was consulted (GMT). Because of the high negative predictive value of the MISS screener, in patients with a negative screener no extensive interview was performed.

Migraine diagnoses were divided into two subtypes: (1) migraine with aura; and (2) migraine without aura. Patients who reported both migraine with and without aura were included in the migraine with aura group. Patients with aura without (migrainous) headache were considered to have migraine with aura.

### 2.3 Assessment of risk factors

Possible vascular risk factors for migraine, stroke and UIA, such as smoking and hypertension, were retrieved from medical records. The presence of these risk factors was also asked during the telephone interview. Smokers were categorized into three groups: never smoked, former smokers, and current smokers. Hypertension was defined as a history of antihypertensive therapy or a blood pressure ≥140/90 mmHg at the outpatient clinic.

### 2.4 Statistical analysis

Univariable logistic regression was used to calculate odds ratios (OR) of migraine history with 95% confidence intervals (CI) between (1) patients with an UIA and controls; and (2) patients with an UIA and patients with a history of TIA or stroke. In multivariable analyses, adjustments were made for age, sex, smoking and hypertension. ORs were calculated for migraine in general and for migraine with aura and migraine without aura separately. A separate analysis was done that excluded UIA patients with suspected stroke as a reason for imaging.

## 3 Results

### 3.1 Patients

**Figure 1:**
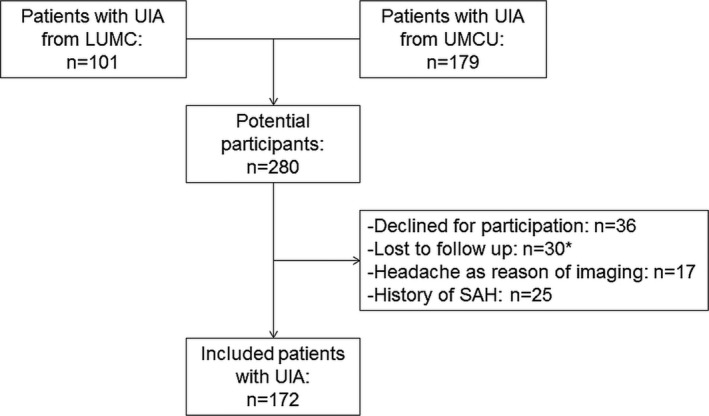
Flowchart of UIA patients. UIA, unruptured intracranial aneurysm; aSAH, aneurysmal subarachnoid hemorrhage. *Loss to follow‐up mostly occurred because patients moved to another city or changed their phone number

We included 172 patients with UIA from the 280 patients with UIA who were eligible to participate in the study (Figure 1). Reasons for exclusion were: decline to participate (*n* = 36), lost to follow up (*n* = 30), history of SAH (*n* = 25), and headache as reason of imaging (*n* = 17). All patients with a history of TIA/stroke from the MISS validation study were included. We included 164 partners of patients as controls: 98 partners of patients with UIA and 66 partners of patients with a history of TIA or stroke. Of all 197 partners of patients with UIA or TIA/Stroke, 33 declined participation in the study. The demographic and clinical characteristics of the included patients and controls are shown in Table 1. Most common reasons of imaging were symptoms of stroke (23.8%), familial aneurysms or SAH (15.1%), preventive total body scan (6.4%) or other (e.g., head injury, meningitis, cranial nerve dysfuntion; 52%).

**Table 1:** Baseline characteristics of the study population

Characteristics	UIA (*n* = 172)	TIA/Stroke (*n* = 221)	Controls (*n* = 164)
Mean age, year (*SD*)	64.2 (10.1)	67.4 (12.3)	63.4 (9.8)
Men (%)	64 (37.2)	117 (52.9)	72 (43.9)
Migraine history (%)	42 (24.4)	49 (22.2)	24 (14.6)
MO (%)	28 (16.3)	28 (12.7)	16 (9.8)
MA (%)	14 (8.1)	21 (9.5)	8 (4.9)
Age onset migraine (*SD*)	23.8 (8.9)^a^		
Smoking status^b^ (%)			
Former smokers	90 (52.3)	92 (41.6)	76 (46.3)
Current smokers	46 (26.7)	35 (15.8)	27 (16.5)
Never smoked	36 (20.9)	90 (40.7)	61 (37.2)
Hypertension (%)	95 (55.2)	134 (60.6)	57 (34.8)

UIA, unruptured intracranial aneurysm; MO, migraine without aura; MA, migraine with aura; aSAH, aneurysmal subarachnoid hemorrhage.

a Data of one person missing.

b Data of four TIA/Stroke patients unavailable.

### 3.2 Migraine and UIA

A lifetime diagnosis of migraine was made in 42 patients of the UIA group (24.4%), in 49 patients of the TIA/stroke group (22.2%), and in 24 of the controls (14.6%). Migraine with aura was diagnosed in 14 patients of the UIA group (8.1%), 21 patients in the TIA/stroke group (9.5%), and 8 controls (4.9%).

Results of the univariable and multivariable analyses are shown in Table 2. In the univariable analysis, OR for migraine in UIA patient versus controls was 1.9 (95% CI 1.1–3.3). The strength of the relationship did not differ between patients with migraine without aura (OR 1.8; 95% CI 0.9–3.5), and patients with migraine with aura (OR 1.7; 95% CI 0.7–4.2). After adjustments for age, sex, hypertension and smoking, the OR for migraine attenuated slightly to 1.7 (95% CI 1.0–3.1; Table 2). In a separate analyses that excluded the 41 UIA patients with suspicion of stroke as reason for imaging, the results were essentially the same (UIA vs. controls; OR 1.8; 95% CI 1.0–3.2 in the univariable analysis and OR 1.6; 95% CI 0.8–3.0 in the multivariable analysis).

**Table 2:** Univariable and multivariate analysis

	Univariable analysis OR (95% CI)		Multivariable analysis^a^ OR (95% CI)	
	UIA versus controls	UIA versus TIA/Stroke	UIA versus controls	UIA versus TIA/Stroke
Migraine total	1.9 (1.1–3.3)	1.1 (0.7–1.8)	1.7 (1.0–3.1)	0.9 (0.5–1.5)
MO	1.8 (0.9–3.5)	1.3 (0.8–2.4)	1.7 (0.8–3.4)	1.3 (0.7–2.4)
MA	1.7 (0.7–4.2)	0.8 (0.4–1.7)	1.5 (0.6–3.9)	0.5 (0.2–1.1)

UIA, unruptured intracranial aneurysm; OR, odds ratio; CI, confidence interval; MO, migraine without aura; MA, migraine with aura.

a After adjustments for age, sex, hypertension and smoking.

## 4 Discussion

Our results suggest that migraine prevalence might be higher in patients with UIA than in controls. The prevalence was comparable with that in the group of patients with TIA or ischemic stroke, who are known from previous studies to have an increased migraine prevalence.

In another retrospective study, an increased prevalence of migraine without aura was found in patients with an intracranial aneurysm in the year before aneurysm rupture (*n* = 177) or detection of an unruptured aneurysm (*n* = 22; Lebedeva, Gurary, Sakovich, & Olesen, 2013). In that study, patients reported a one year prevalence of migraine (migraine with or without aura) of 40.2% compared with 8.8% in controls. Our study revealed a lower migraine prevalence in the UIA group, which may be explained by difference in study population since aSAH patients were excluded from our study. A history of SAH may lead to a recall bias by overestimating or misinterpreting previous headache complaints by patients. In addition, cognitive impairment after aSAH may have had an influence on the interviews in these patients.

Of all patients with UIA who were included in our study, 110 were derived from another study on risk factors for UIA (Vlak et al., 2013). This other study investigated a range of possible risk factors by sending out questionnaires by mail, which included only one question on migraine history. To achieve a more reliable and uniform assessment of migraine history, we decided to approach all patients again for the present study by telephone with a short validated migraine screener followed by an extensive interview. With our validated migraine assessment we found a remarkably higher prevalence of migraine in the overlapping group of 110 UIA patients (24.4% in our study vs. 7% in the previous study; Vlak et al., 2013). This difference may be due to an underestimation of migraine prevalence by only asking whether patients had ever been diagnosed with migraine. Many migraine patients may never have consulted a physician for their headache complaints, and have, therefore, never been diagnosed with migraine.

Some limitations of our study should be acknowledged. First, selection bias may have occurred, as some patients with UIA were diagnosed after having an ischemic stroke. Because migraine is an established risk factor for an ischemic stroke, this could have influenced our results. However, after excluding all patients with a suspected stroke as reason for detecting the UIA, the results of our analysis were comparable with the main analysis. The diagnosis TIA was made by a stroke neurologist based on clinical grounds. Although the neurologists in our department are specialized in both vascular neurology and migraine, we cannot completely rule out that some migraine auras might have been misdiagnosed as TIA. Second, all participants were informed that our study was about migraine. Therefore, patients with migraine complaints may have been more likely to respond and participate in our study than patients without migraine. However, as patient and control groups were all approached in the same way, we consider it unlikely that this selection bias has greatly influenced our results. Third, the mean age of all participants was approximately 65 years, and as the peak prevalence of migraine is around 35 years, some of the migraine diagnoses were based on complaints occurring in the past (Ferrari, 1998). Although the time between interview and migraine complaints is similar on an average for all groups, the patient groups may have been reflecting more intensely on previous health problems since their diagnosis, causing recall bias. Fourth, because we performed a case–control study we could not infer any causal relations. Ideally, a cohort study should be performed, but such a study is difficult to carry out in practice, because it requires large groups of persons with and without migraine with long‐term brain imaging follow‐up due to the low absolute prevalence of intracranial aneurysms. Fifth, 26 patients with UIA were diagnosed after screening because of familial UIA. Migraine could also be familial, which could result in an overestimation of the effect. Of the 26 patients with familial UIA, only four were relatives from two different families and none of them were diagnosed with migraine. Therefore, it is unlikely that this have influenced our results. Last, we could not investigate the relation between migraine diagnosis and size of the aneurysm because information about the size of the aneurysms in our study was lacking.

There are also strong points of our study. We had access to a large number of patients with UIA, and were not only able to compare them with controls but also with patients with a history of TIA or stroke. We used partners of patients as controls to make it less likely that enviromental factors or vascular risk factors for UIA would differ between the cases and the controls. In addition, all interviews in patients with UIA and controls were performed by the same interviewer based on the ICHD criteria, preventing any inter‐interviewer differences.

The pathway through which migraine could be related to UIA is unclear and probably multifactorial. One hypothesis is that an UIA itself can cause migraine. Although it is generally thought that unruptured aneurysms do not cause headache, it has been hypothesized that input from a perivascular sensory nerve terminal around the terminals or a local stimulator of the dura mater, may increase the sensitization in the cranial nervous system (Lebedeva et al., 2013). A previous study found no difference in the prevalence of migraine without aura between patients with giant aneurysms and patients with small aneurysms (Lebedeva et al., 2013). Moreover, the mean age at onset of migraine in our group was around 24 years, which suggests that migraine most likely started before the development of the UIA, although we do not know the age at which these arose.

Another hypothesis is that migraine may influence the development of UIA. Cortical spreading depression, a presumed core mechanism in migraine pathophysiology, is thought to activate the trigeminovascular pathway, leading to the release of vasoactive peptides (Noseda & Burstein, 2013). Prolonged exposure to these vasoactive peptides may affect vessel wall integrity and facilitate development of UIA in susceptible individuals, but this is still a speculative theory. Also, endothelial dysfunction has been suggested in patients with migraine ( Lee et al., 2008; Yetkin, Ozisik, Ozcan, Aksoy, & Turhan 2006; Yetkin, Ozisik, Ozcan, Aksoy, & Turhan, 2007). Furthermore, genetic studies showed that a SNP in the LRP1 protein, that plays a role in vascular wall integrity, increases the risk of both migraine without aura and abdominal aneurysms (Bown et al., 2011; Freilinger, Anttila, De Vries, & Malik, 2013). The association between LRP1 and intracranial aneurysms has to our knowledge not been studied yet. Apart from the hypothesized direct causal associations, it is likely that there are common cardiovascular risk factors associated with both migraine and UIA, such as hypertension and smoking although we adjusted for these factors in our analyses (Scher et al., 2005; Vlak et al., 2013). More studies are needed to confirm our findings and to further investigate the underlying pathophysiology between the possible association between migraine and intracranial aneurysms.

## Conflict of Interest

On behalf of all authors, the corresponding author states that there is no conflict of interest.

## Disclosures

E.H. Witvoet: none; Pelzer reports support for conference visits from Menarini and Allergan UK.; Terwindt reports grants and consultancy/industry support from Merck and Menarini, and independent support from NWO; Rinkel: none; Algra: none; Vlak: none; M.J.H. Wermer: none.
